# Case Report: Dynamic characteristics of myocardial depression after severe bupivacaine-induced local anesthetic systemic toxicity

**DOI:** 10.3389/fcvm.2026.1896055

**Published:** 2026-07-29

**Authors:** Shujian Zhang, Bingjie Lai, Yang Hu, Fuxi Ji, Qi Yang, Yongjie Yin, Jiakun Tian, Debiao Song

**Affiliations:** Department of Emergency and Critical Care Medicine, Second Hospital of Jilin University, Changchun, Jilin, China

**Keywords:** bedside echocardiography, bupivacaine, cardiogenic shock, local anesthetic systemic toxicity (LAST), myocardial depression, venoarterial extracorporeal membrane oxygenation, (VA-ECMO)

## Abstract

**Background:**

Bupivacaine-induced local anesthetic systemic toxicity (LAST) can lead to severe myocardial depression and circulatory collapse, and some patients may require venoarterial extracorporeal membrane oxygenation (VA-ECMO) support. However, serial and visually interpretable clinical data regarding the dynamic changes in cardiac function, the timing of the nadir, and the recovery trajectory of severe bupivacaine-induced myocardial depression remain limited.

**Case presentation:**

A 32-year-old woman at term underwent combined spinal-epidural anesthesia. Shortly after intrathecal administration of 2.0 mL of 0.5% bupivacaine, she developed sacrococcygeal numbness, limb tremors, and bilateral lower-limb convulsions, followed by rapid progression to severe circulatory collapse. Bedside echocardiography revealed markedly impaired left ventricular systolic function, with a left ventricular ejection fraction (LVEF) of approximately 27%. Despite treatment with lipid emulsion and vasoactive agents, hemodynamic stability could not be maintained, and VA-ECMO support was therefore initiated. Serial bedside echocardiography showed that cardiac function reached its nadir 24 h after symptom onset, with a stroke volume of 10.4 mL and an LVEF of 13.2%. Cardiac function then remained at a low-level plateau for the following several days and showed marked recovery approximately 120 h after onset, meeting the criteria for ECMO weaning. After decannulation, cardiac function continued to improve, and the patient was successfully extubated and transferred to the obstetrics department for further management.

**Conclusions:**

Severe bupivacaine-induced myocardial depression may be characterized by a delayed functional nadir followed by a reversible recovery process. VA-ECMO can provide effective circulatory support for cardiogenic shock refractory to conventional therapy. Serial bedside echocardiography may help assess the trajectory of cardiac recovery and guide the timing of ECMO weaning.

## Introduction

Bupivacaine is a long-acting amide local anesthetic widely used in clinical practice; however, inadvertent intravascular administration can precipitate local anesthetic systemic toxicity (LAST) (1). Severe LAST can rapidly involve the central nervous and cardiovascular systems, resulting in neurological symptoms, malignant arrhythmias, circulatory collapse, and even cardiac arrest (2, 3). Although 20% lipid emulsion therapy and advanced life support remain key therapeutic interventions (4–6), some patients may still develop persistent myocardial depression. In this setting, venoarterial extracorporeal membrane oxygenation (VA-ECMO) may serve as a circulatory support strategy to bridge patients to myocardial recovery (4, 7).

Previous case reports have described successful resuscitation of bupivacaine-associated LAST with lipid emulsion therapy or extracorporeal membrane oxygenation (ECMO). However, most published reports have primarily focused on the resuscitative measures and final outcomes (8–11). Serial echocardiographic data describing the dynamic changes in cardiac function, the timing of the functional nadir, and the subsequent recovery trajectory during ECMO support in patients with severe bupivacaine-induced cardiotoxicity remain limited.

Here, we report a case of severe LAST caused by inadvertent intravascular administration of bupivacaine. The patient developed circulatory collapse predominantly characterized by profound myocardial depression, and effective circulation could not be maintained despite conventional therapy; VA-ECMO support was therefore initiated. Notably, serial echocardiography in this case provided a complete record of left ventricular functional changes: SV declined to a nadir of 10.4 mL 24 h after symptom onset, subsequently remained at approximately 25–28 mL, and increased to 40.1 mL at approximately 120 h after onset, corresponding to illness day 6, at which point the patient met the criteria for ECMO weaning. This case may provide a useful reference for dynamic cardiac function monitoring, assessment of recovery trends, and selection of the optimal weaning time during ECMO support in such critically ill patients. It also suggests that severe bupivacaine-induced myocardial depression may be characterized by a delayed functional nadir followed by a reversible recovery process.

## Case presentation

The patient was a 32-year-old woman at 38 weeks of gestation. She had undergone regular prenatal examinations during pregnancy, with no apparent abnormalities. Preoperative assessment showed that she was conscious and alert, had no history of underlying disease, and had stable vital signs. She was classified as American Society of Anesthesiologists physical status II, with New York Heart Association functional class I.

At 07:00 on April 1, 2026, the patient was transferred to the operating room. At 07:20, combined spinal-epidural anesthesia was performed at the L2–3 interspace. A total of 2.0 mL of 0.5% bupivacaine was injected into the subarachnoid space, and the epidural catheter was inserted 3 cm into the epidural space. At 07:30, the patient reported sacrococcygeal numbness and developed limb tremors. After removal of the epidural catheter, her symptoms did not improve significantly. At 07:35, she developed convulsions of the left lower limb, which rapidly progressed to bilateral lower-limb convulsions accompanied by agitation. General anesthesia was therefore initiated. The operation was completed at 08:00. Based on her clinical manifestations, LAST was suspected. She was treated with 105 mL of 20% lipid emulsion, followed by continuous infusion at 0.25 mL/kg/min. At that time, her blood pressure remained stable; however, her heart rate persisted at 130–140 beats/min, and convulsive movements involving the abdomen and both lower limbs were still observed. She was therefore transferred to the intensive care unit (ICU) for further management.

**Figure 1:**
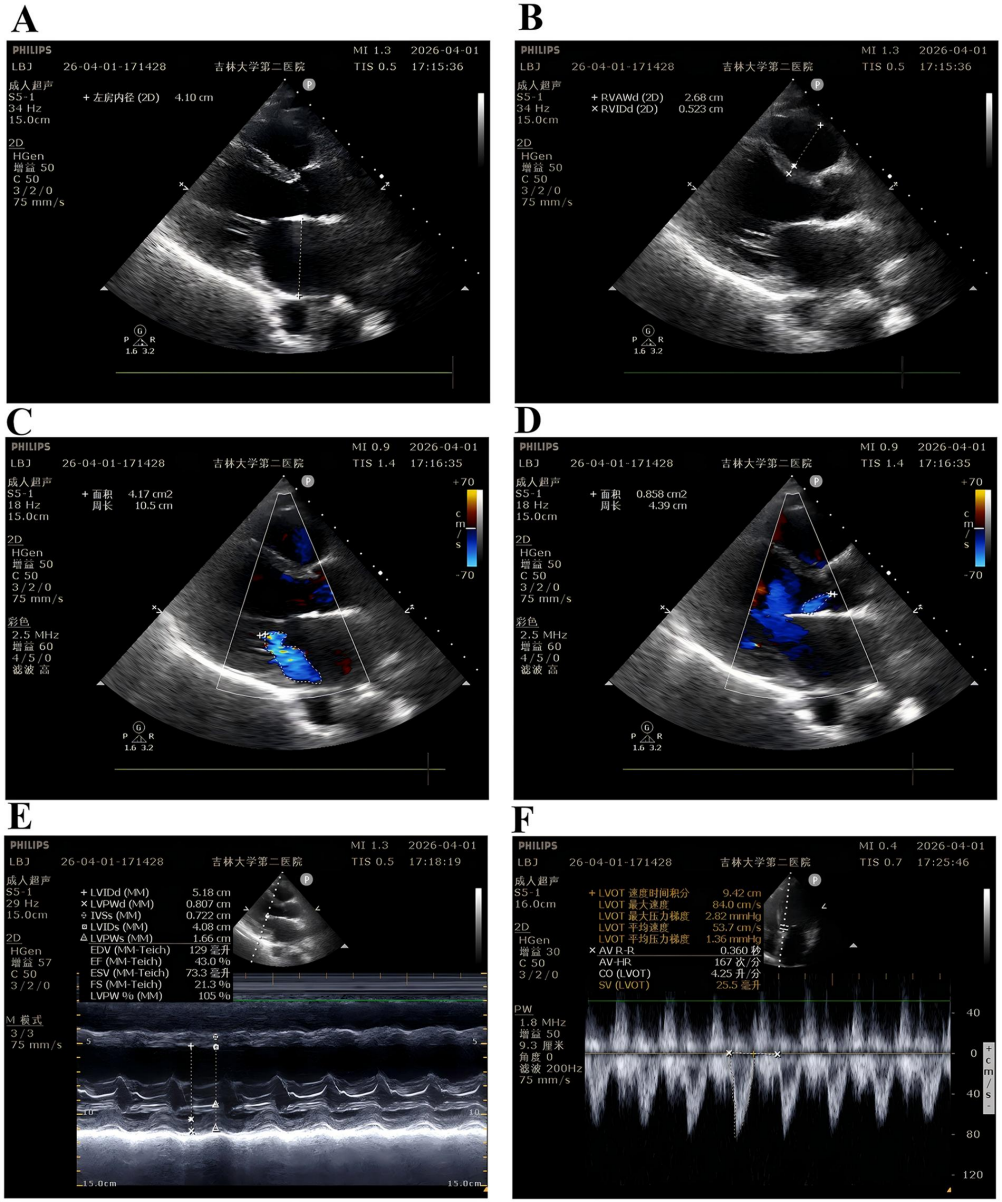
Bedside transthoracic echocardiographic findings before initiation of ECMO support. **(A)** Parasternal long-axis view showing measurement of the left atrial anteroposterior diameter. **(B)** Parasternal long-axis view showing measurement of right ventricular anterior wall thickness and right ventricular end-diastolic diameter. **(C)** Color Doppler imaging showing mitral regurgitation and measurement of the regurgitant jet area. **(D)** Color Doppler imaging showing aortic regurgitation and measurement of the regurgitant jet area. **(E)** M-mode echocardiography showing measurement of left ventricular dimensions, with LVEF calculated using the Teichholz method. **(F)** Pulsed-wave Doppler of the left ventricular outflow tract showing measurement of the velocity-time integral, with calculation of SV and CO.

After admission to the ICU, the patient's condition deteriorated rapidly. Physical examination showed that both pupils were equal and round, with a diameter of approximately 6 mm, and pupillary light reflexes were absent. Her heart rate fluctuated between 140 and 170 beats/min. Blood pressure decreased markedly within 1 h after ICU admission and rapidly progressed to refractory circulatory failure, requiring norepinephrine at 0.55 μg/kg/min, epinephrine at 0.09 μg/kg/min, and low-dose vasopressin to maintain circulation. The 12-lead electrocardiogram showed a heart rate of 153 beats/min and nonspecific ST-segment abnormalities in multiple leads (Supplementary Figure 1). Laboratory tests showed an elevated high-sensitivity cardiac troponin level of 11,023 pg/mL (reference range, 0–17.5 pg/mL), myoglobin of 245.3 ng/mL (reference range, 17.4–105.4 ng/mL), and creatine kinase-MB of 11.1 ng/mL (reference range, 0.6–6.3 ng/mL). A venous blood sample was simultaneously obtained and sent to an external laboratory for plasma bupivacaine concentration measurement, which showed a level of 2.5 μg/mL. Cranial computed tomography and magnetic resonance imaging revealed no acute abnormalities. Bedside echocardiography demonstrated left heart enlargement, diffusely reduced left ventricular wall motion in all segments except the apical region, markedly impaired left ventricular systolic function with an LVEF of approximately 27%, mild-to-moderate mitral regurgitation, and mild aortic regurgitation (Figure 1). Taken together, these findings indicated severe circulatory failure, and VA-ECMO was urgently established for circulatory support.

**Figure 2:**
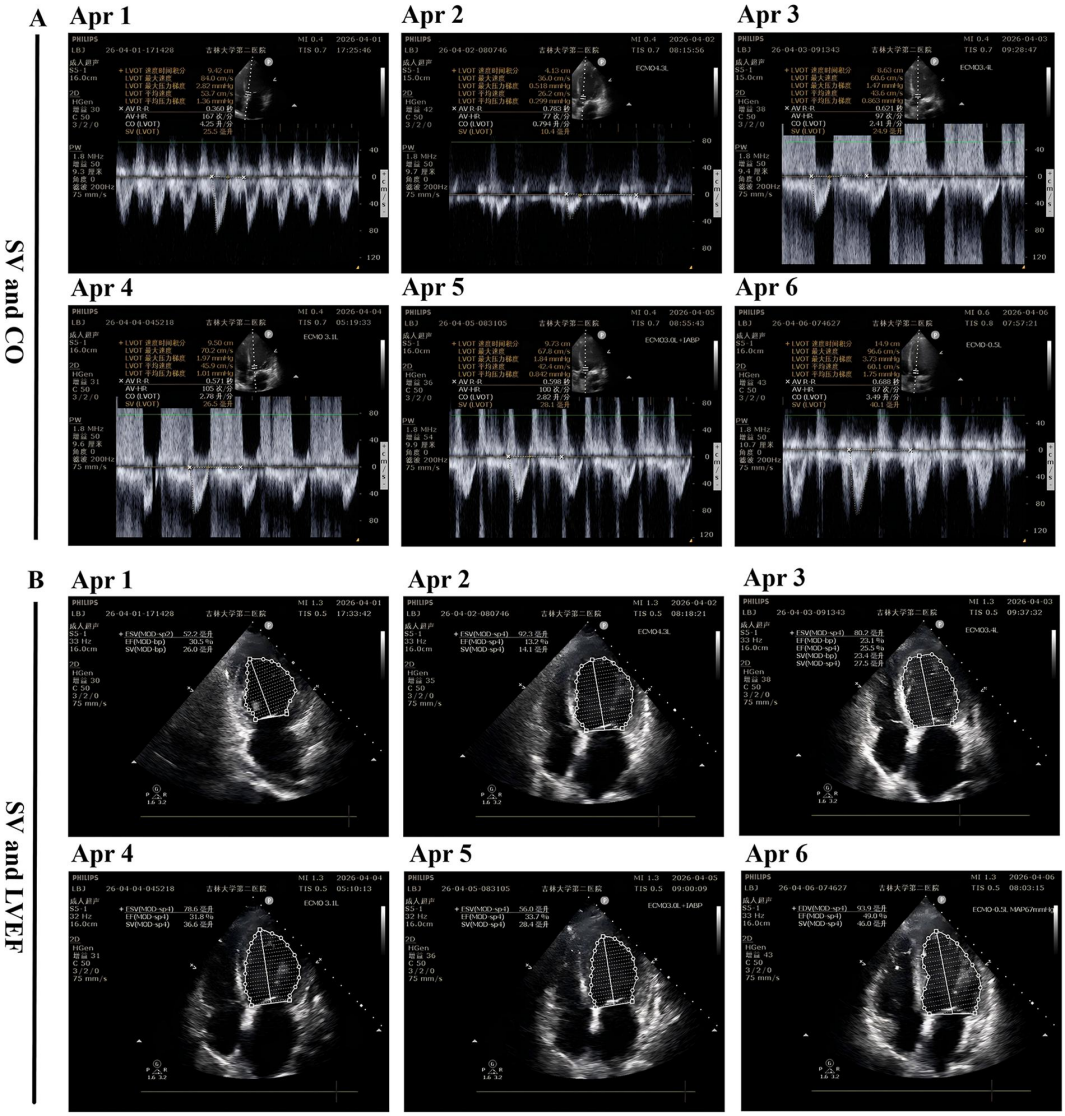
Dynamic bedside echocardiographic assessment of CO and LVEF during ECMO support. **(A)** From April 1 to April 6, the velocity-time integral was measured using pulsed-wave Doppler of the left ventricular outflow tract, and SV and CO were calculated. **(B)** From April 1 to April 6, left ventricular volumes were measured using the single-plane modified Simpson method in the apical four-chamber view, and SV and LVEF were calculated.

After initiation of VA-ECMO support, coronary angiography showed no significant coronary artery stenosis or obstructive lesions. Thereafter, bedside echocardiography was performed daily to assess changes in cardiac function (Figure 2). At 24 h after symptom onset, SV decreased to 10.4 mL, cardiac output (CO) was 0.79 L/min, and LVEF was 13.2%. During the following several days, SV remained at 25–28 mL, CO at 2.4–2.8 L/min, and LVEF at 23%–33%. At approximately 120 h after symptom onset, corresponding to illness day 6, SV increased to 40.1 mL, CO to 3.49 L/min, and LVEF to 49%. Meanwhile, vasoactive agents had been tapered and discontinued. After assessment with a pump-controlled retrograde flow trial, the patient was successfully weaned from VA-ECMO.

**Figure 3:**
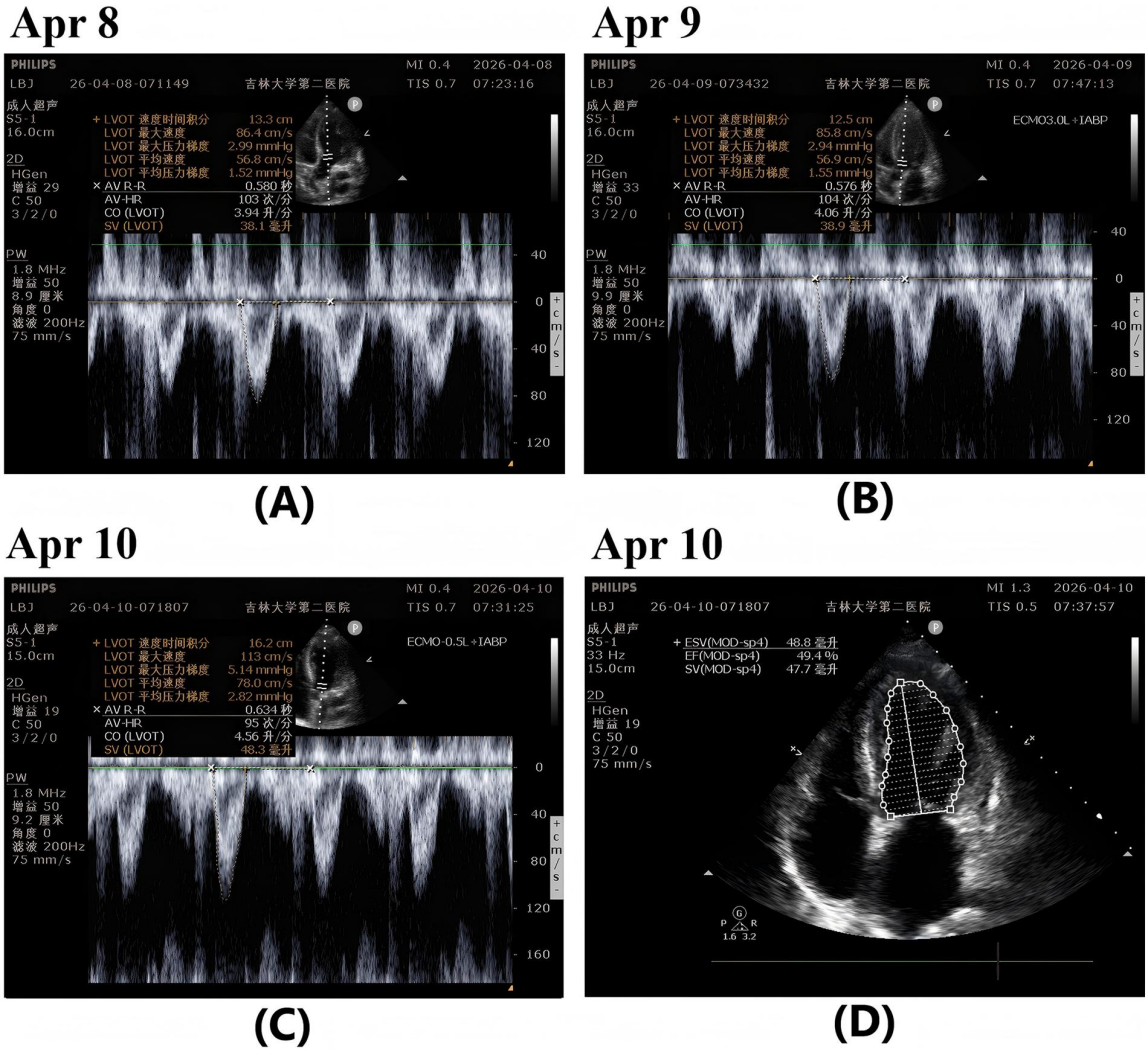
Serial bedside echocardiographic assessment of cardiac function after ECMO weaning. **(A–C)**: From April 8 to April 10, the velocity-time integral was measured using pulsed-wave Doppler of the left ventricular outflow tract, and SV and CO were calculated. **(D)** On April 10, left ventricular volumes were measured using the single-plane modified Simpson method in the apical four-chamber view, and LVEF was calculated.

After decannulation, cardiac function continued to be assessed daily (Figure 3). SV and CO increased steadily, and cardiac function gradually recovered. On April 10, 2026, SV was 48.3 mL, CO was 4.56 L/min, and LVEF was 49.4% (Figure 3). Invasive mechanical ventilation was therefore discontinued, and the endotracheal tube was removed. After extubation, the patient was conscious, with stable vital signs, and was transferred to the obstetrics department the following day for further treatment.

## Discussion

Bupivacaine is a long-acting amide local anesthetic widely used in clinical practice; however, it has substantial cardiovascular toxicity and may rapidly induce severe LAST after entering the systemic circulation (1). In the present case, the patient developed neurological symptoms shortly after intrathecal administration of bupivacaine and rapidly progressed to circulatory collapse. An early 12-lead electrocardiogram showed nonspecific ST-segment abnormalities in multiple leads, suggesting ischemia-like changes or repolarization abnormalities. Previous studies have indicated that similar ST–T changes are not specific to local anesthetic systemic toxicity and may also occur in fulminant myocarditis, hypokalemia, and drug-induced repolarization abnormalities (12–14). In addition, because bupivacaine exerts sodium channel-blocking effects, Brugada phenocopy or Brugada-like electrocardiographic changes should also be considered in the differential diagnosis (15). However, the patient had no clear infectious prodromal symptoms before onset, and the electrocardiogram showed no typical coved-type ST-segment elevation in the right precordial leads. The electrolyte findings and medication history also did not support hypokalemia or other drug-related ST–T abnormalities as the primary cause. In contrast, there was a close temporal relationship between the intrathecal injection of bupivacaine and symptom onset, with neurological manifestations occurring first, followed by rapid progression to severe circulatory failure. Therefore, based on the timing of drug administration, clinical manifestations, and electrocardiographic findings, bupivacaine-associated local anesthetic systemic toxicity was considered the most likely clinical diagnosis in this case (2, 3, 16). Administration of 20% lipid emulsion is a core treatment for severe LAST (4–6). However, effective circulation could not be maintained in this patient despite lipid emulsion therapy and advanced life support; therefore, VA-ECMO was initiated as circulatory support to bridge the patient to myocardial recovery. This approach is also consistent with guideline recommendations that extracorporeal circulatory support may be considered for toxic cardiogenic shock refractory to conventional therapy (4, 7).

Although cardiac function eventually recovered under VA-ECMO support in this case, the more important clinical value lies in the serial bedside echocardiographic documentation of the dynamic recovery process of bupivacaine-induced toxic myocardial depression. Because reversible myocardial depression can occur in various clinical settings, and because the echocardiographic recovery patterns may differ according to etiology, it is necessary to compare the present case with other common forms of reversible myocardial injury. In a recent study, Jha et al. used serial echocardiographic follow-up and found that patients with Takotsubo syndrome and anterior ST-segment elevation myocardial infarction (STEMI) both showed partial recovery of regional wall motion within 72 h, whereas improvement in left ventricular function could continue beyond day 7 (17). These findings suggest that myocardial stunning in these conditions is characterized by early initiation of recovery followed by sustained improvement. Myocardial dysfunction after resuscitated cardiac arrest is usually most pronounced in the early post-resuscitation period and typically improves or recovers within approximately 72 h (18, 19).

In contrast, bupivacaine-associated toxic myocardial depression in the present case showed a distinct echocardiographic trajectory, characterized by a nadir at 24 h, a plateau from days 2–5, and marked recovery beginning on day 6. This pattern differs from the rapid improvement commonly observed within 72 h after cardiac arrest and from the early, gradual improvement typical of myocardial stunning after reperfused STEMI. A similar recovery trajectory is not unique to bupivacaine toxicity. In patients with venlafaxine poisoning, LVEF may decrease to <20% or even <10% approximately 20–24 h after ingestion, followed by successful VA-ECMO weaning around day 5 (20, 21). Patients with bupropion overdose may also progress to severe myocardial depression within approximately 24 h, with an initial LVEF as low as 9%, and recover to 53% by day 4 after mechanical circulatory support (22). In a patient with acute cardiotoxicity related to 5-fluorouracil, LVEF further decreased to <10% at 24 h after admission, recovered to 45% by day 4 under ECMO support, and ECMO was discontinued on day 5 (23). These reports, together with the present case, suggest that some forms of drug-induced toxic myocardial depression do not improve immediately after circulatory support is established, but may instead undergo a dynamic process of early deterioration, a transient plateau, and gradual recovery over the following days. Serial bedside echocardiography may facilitate dynamic assessment of the trajectory of myocardial functional recovery and provide a reference for determining the timing of VA-ECMO weaning. However, echocardiography has limited ability to assess tissue-level abnormalities such as myocardial edema, inflammation, necrosis, or fibrosis. Therefore, after the acute phase, cardiac magnetic resonance imaging may serve as an important complementary examination to differentiate myocarditis, Takotsubo syndrome, ischemic myocardial injury, and reversible myocardial depression associated with drug-induced cardiotoxicity, as well as to further evaluate the presence of residual myocardial injury (24, 25).

This case has several limitations. First, the data were derived from a single patient, and a fixed time window for cardiac function recovery after severe bupivacaine-associated LAST cannot be determined from this case alone. Second, VA-ECMO support alters cardiac preload and afterload; therefore, echocardiographic parameters should be interpreted in conjunction with ECMO flow, vasoactive drug dosage, and the overall hemodynamic status.

## Conclusion

Myocardial depression caused by severe bupivacaine cardiotoxicity may have a delayed functional nadir followed by a reversible recovery process. Serial bedside echocardiography can provide a direct visualization of its dynamic evolution.

## Data Availability

The original contributions presented in the study are included in the article/Supplementary Material, further inquiries can be directed to the corresponding authors.
